# Site-Specific and Fluorescently Enhanced Installation of Post-Translational Protein Modifications via Bifunctional Biarsenical Linker

**DOI:** 10.1021/acsomega.4c05828

**Published:** 2024-10-30

**Authors:** Anastasiia Antonenko, Adam Pomorski, Avinash Kumar Singh, Katarzyna Kapczyńska, Artur Krężel

**Affiliations:** † Department of Chemical Biology, Faculty of Biotechnology, University of Wroclaw, Joliot-Curie 14a, 50-383 Wrocław, Poland; ‡ Department of Immunology of Infectious Diseases, Hirszfeld Institute of Immunology and Experimental Therapy, Polish Academy of Sciences, 53-114 Wrocław, Poland; § Department of Laboratory Medicine and Pathology, Mayo Clinic, Rochester 55901, Minnesota, United States

## Abstract

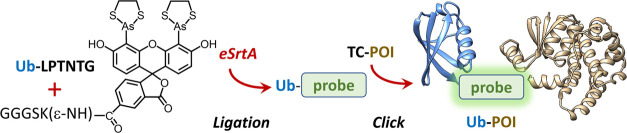

To understand how particular post-translational modifications (PTMs) affect the function of a target protein, it is essential to first prepare and investigate the target with the modification at the desired position. This drives the continuous development of site-specific protein modification technologies. Here, we present the chemical synthesis and application of the biarsenical linker SrtCrAsH-EDT_2_, which has a dual labeling functionality. This linker, containing a sortase A recognition motif, can be conjugated with any protein containing the LPXTG motif at the C terminus, such as ubiquitin and the SUMO tag, and then attached to a protein of interest (POI) containing a terminal or bipartite (intramolecularly placed) tetracysteine motif. This modification of the POI facilitates the straightforward and rapid incorporation of PTMs, which are further highlighted by the fluorescent biarsenical probe. Consequently, this directly correlates proteins’ physical properties and cellular roles under various physiological conditions or in disease states. The proposed one-pot labeling methodology can be utilized to explore the effects of PTMs on proteins, affecting their structure, function, localization, and interactions within the cellular environment. Understanding these effects is crucial for uncovering the complex mechanisms that regulate cellular function and dysfunction.

## Introduction

Methods for site-specific modification of proteins remain in high demand.^1,2^ They are utilized to introduce various effectors to target biomolecules, e.g., for imaging reagents.^3^ However, one of the most prominent applications is related to the introduction of post-translational modifications (PTMs). They influence protein structure and function, and PTM dysregulation is implicated in various diseases.^4−6^ However, to truly understand their biological relevance, these modifications have to be specifically placed at selected locations within the protein sequence. PTMs can be roughly divided into two categories by their size. First is a modification with a small chemical group, e.g., phosphorylation or acetylation.^4^ The second is the covalent attachment of proteins, e.g., ubiquitinylation or SUMOylation.^4^ Over the years, numerous methods of site-specific PTM introduction have been developed.^7,8^ Some of them rely on a modification using an expansion of the genetic code or activity of a protein ligase, e.g., sortase.^9−11^ These methods are pivotal for modifying proteins in a controlled and specific manner, which is essential for research, diagnostics, and therapeutic applications. However, most protein ligase-mediated site-specific approaches rely on recognition sequences that are located only on protein termini. Though some modifications, e.g., acetylation or ubiquitination, can be located on the N-terminus, they are usually found to modify amino acid residues within the sequence of proteins.^12^ On the other hand, the efficiency of the genetic code expansion to encode an amino acid derivative with click-chemistry moiety can be very limited.^13^ The key to developing a method to introduce a PTM site specifically is utilizing the shortest possible recognition sequence. In this way, the introduction of a new sequence would not disturb the protein structure or function. One such successful system is a tetracysteine (TC) tag and its selective biarsenical binders, e.g., FlAsH-EDT_2_ (fluorescein arsenical hairpin) originally developed by the Tsien group.^14,15^ There are numerous reports where the addition of the CCXXCC tag did not result in an alteration of protein activity.^16^ Biarsenical fluorescent probes are not only useful as fluorescent protein alternatives for localizing proteins within cells, but also for studying various aspects of protein function such as folding, degradation, activity control, singlet oxygen inactivation, oligomerization, and protein purification, depending on their specific chemical properties.^16^ Specifically to our application, biarsenical probes were previously used to link proteins into homodimers.^17^ Moreover, the probes are compatible with sortase-based protein ligation, which has garnered significant interest in biotechnology and bioconjugation applications.^18−20^ Sortase-mediated protein ligation is a robust, specific, and simple-to-execute technique that can be broadly used for the specific conjugation of proteins with polypeptides or molecules that possess distinct biochemical and biophysical characteristics.^21^ Sortase enzymes can be found in certain bacteria, primarily Gram-positive species.^22^ Their primary function is to catalyze the covalent attachment of surface proteins to the peptidoglycan cell wall. This mechanism is crucial for various bacterial processes, including adhesion to host tissues, biofilm formation, and immune evasion.^22^ The enzymatic reaction catalyzed by sortase enzymes involves a two-step mechanism: the active-site cysteine residue of sortase attacks the carbonyl carbon of the threonine residue within the conserved LPXTG motif (where X can be any amino acid), forming a thioacyl intermediate. Then, it is resolved by nucleophilic attack from the amino group of a cross-linking glycine residue within the peptidoglycan, resulting in the covalent attachment of the substrate protein to the cell wall.^23^ Thus, while a single glycine can act as a nucleophile, longer chains of oligoglycine lead to better yields and more effective enzymatic reactions.^24^ Researchers have utilized sortase-mediated ligation for various applications, including protein labeling,^20,25,26^ cyclization,^27^ immobilization,^28^ protein–protein fusion,^29^ and the generation of multifunctional protein constructs.^30−32^

Here, we present the chemical synthesis and application of biarsenical SrtCrAsH-EDT_2_, a derivative of a 5-carboxyfluorescein-based biarsenical probe (5-CrAsH-EDT_2_) with a dual labeling functionality. This probe containing sortase A recognition motif can be conjugated with any protein containing LPXTG motif at C terminus, here ubiquitin (Ub) and SUMO tag and then attached to the protein of interest (POI) containing terminal, internal or bipartite (intramolecularly placed) tetracysteine motif. Such decoration of POI results in the simple and fast installation of PTM that is illuminated by a fluorescent CrAsH-based biarsenical platform. This probe brings new possibilities in designing bifunctional molecules to study the role of the PTM modifications placed at particular sites of a target protein.

## Results and Discussion

### Bifunctional Biarsenical Probe Linker Synthesis

A biarsenical probe with dual function, SrtCrAsH-EDT_2_, was prepared by a combination of solid-phase peptide synthesis (SPPS), solution peptide coupling with biarsenical precursor CrAsH-EDT_2_ followed by product mercuration, transmetalation, and final probe capping with arsenic protectant. For the site-specific modification, we chose *Staphylococcus aureus* sortase A pentamutant (eSrtA), which recognizes the GGG sequence for efficient cleavage and subsequent ligation.^29^ A single glycine is required for attack in the transpeptidation reaction mediated by sortase enzymes. However, the efficiency of the enzyme’s action depends significantly on the length of this oligoglycine chain.

**Figure 1:**
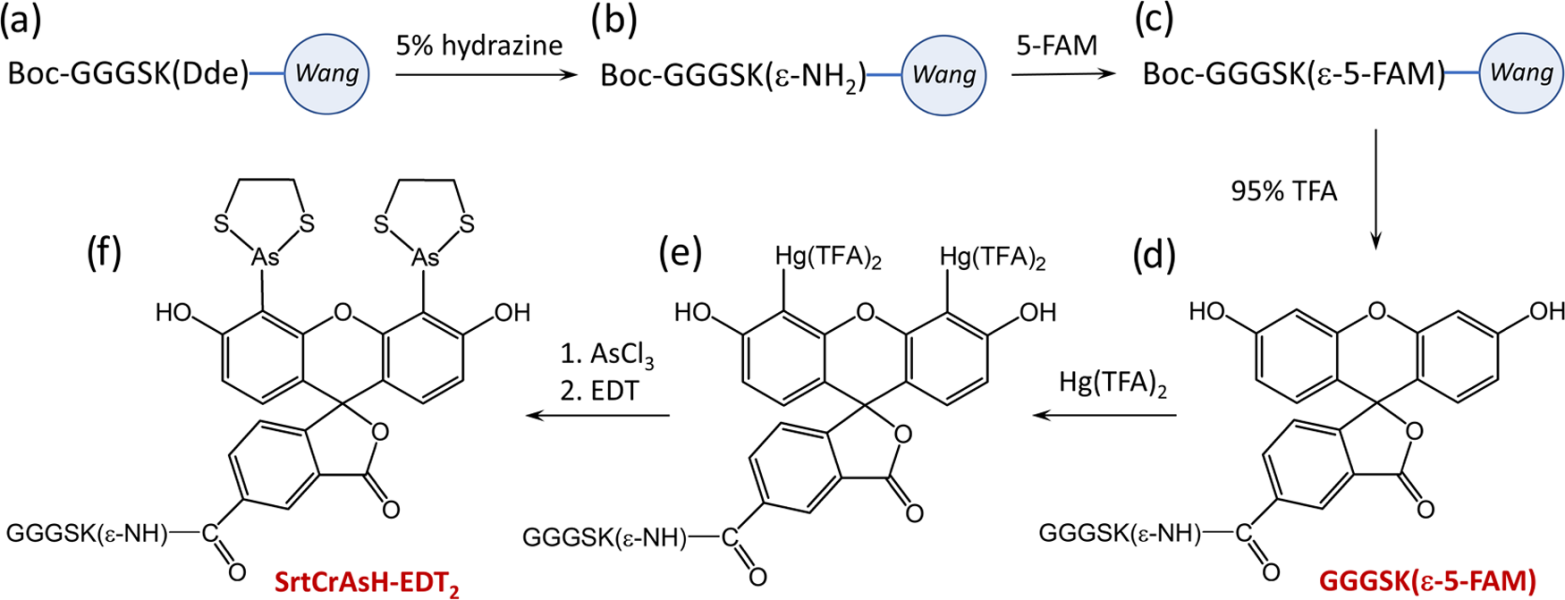
Synthesis of dual-specific biarsenical probe that allows one-pot site-specific incorporation of large post-translational modifications such as SUMOylation and ubiquitinylation utilizing sortase-based ligation. A synthesized peptide with a GGG recognition motif for the sortase is modified utilizing lysine residue with 5-FAM (steps a–d). The fluorescent label on the peptide undergoes mercurization and transmetalation to incorporate arsenic atoms that will bind with high affinity to the tetracysteine motif (steps e and f).

A longer chain, such as a triglycine, provides multiple attack points and more flexibility, potentially leading to a higher yield in the enzyme-mediated attachment process.^24^ Therefore, in the first step of probe linker synthesis utilizing Fmoc-based SPPS, we obtained a peptide with a triglycine motif, GGGSK, N-terminally blocked with the Boc group (Figure 1a). Its lysine residue was blocked with a removable 1-(4,4-dimethyl-2,6-dioxocyclohex-1-ylidene)ethyl (Dde) group, which was selectively deprotected using a 5% hydrazine solution in *N*,*N*-dimethylformamide (DMF) (Figure 1b). The product of that reaction was coupled on a resin with 5-carboxyfluorescein using Oxyma Pure and *N*,*N*′-diisopropylcarbodiimide (DIC) (Figure 1c). The coupling product, GGGSK(ε-5-FAM), cleaved from the resin using a trifluoroacetic acid (TFA) cocktail, was purified using reversed-phase high-performance liquid chromatography (RP-HPLC) and lyophilized (Figure 1d). To confirm the intermediates and product masses’ correctness, they were analyzed by electrospray ionization mass spectrometry (ESI-MS) (Table S1).

In the next step of the synthesis, purified GGGSK(ε-5-FAM) was first reacted with mercury(II) trifluoroacetate in TFA to obtain 4′,5′-substituted 5-FAM with mercury atoms (Figure 1e). We noticed that, in contrast to other biarsenical probes, this reaction does not result in the precipitation of the product under the used conditions. It is due to the presence of the peptide chain at the 5-fluorescein position, which significantly increased the solubility of the probe. As described in the Experimental Procedures, adjustments were made to process the product as a dry powder. Mercurated product dissolved in NMP was transmetalated with arsenic(III) using AsCl_3_ in the presence of palladium(II) acetate as a catalyst.^15^ Reaction was stopped by the addition of phosphate buffer with acetone containing EDT, which caps arsenic atoms protecting them from environment different than the tetracysteine motif (Figure 1f). The standard postreaction workflow includes extraction of the biarsenical product using chloroform. However, the desired product was not found there due to high product polarity. The majority of the product was found in aqueous fraction and was purified without evaporation using HPLC in slightly basic conditions using an NH_4_CO_3_/MeCN gradient. The mass correctness was confirmed by using ESI-MS (Table S1). The dried, purified probe linker was stable for >6 months at −20 °C.

**Figure 2:**
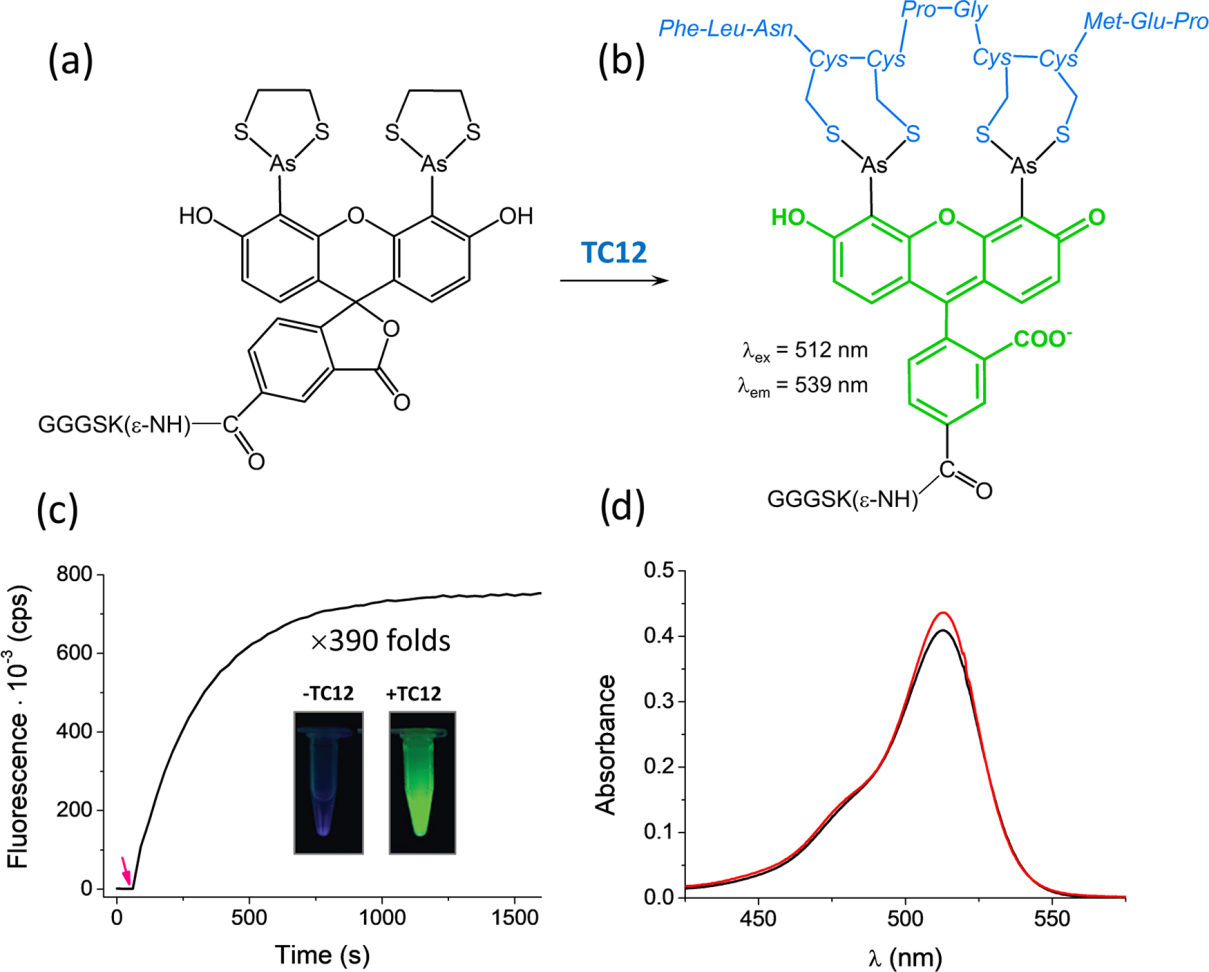
Characterization of the physical properties of the SrtCrAsH linker probe. The scheme (a, b) represents the most important feature of the biarsenical dye, where its fluorescence is quenched prior to the addition of the tetracysteine tag (here TC12). (c) Kinetics of SrtCrAsH-EDT_2_ probe binding to model TC12 peptide. Three times molar excess of TC12 ensures that binding is completed within 25 min. Red arrow indicates addition of the TC12 peptide. We observe an increase of over 390 times in fluorescence. (d) Absorbance spectra of SrtCrAsH-EDT_2_ probe linker as a free dye (black line) and conjugated to TC12. The maximum absorbance is 513 nm, which did not shift upon binding to the model TC12 peptide. We observed only a slight increase in the absorbance of the conjugated SrtCrAsH by 6.6%.

We next characterized the physicochemical properties of the new SrtCrAsH-EDT_2_ probe. As expected for biarsenicals, the fluorescence of the probe is strongly quenched and occurs only after binding to the tetracysteine motif. In this complex, neighboring cysteine residues bind to two arsenic atoms and two EDT caps dissociate (Figure 2a). In the case of SrtCrAsH-EDT_2_, its fluorescence at 539 nm increases over 390 times after the addition of optimized TC12 peptide, NH_2_-FLNCCPGCCMEP-NH_2_ (Figure 2b). Similarly to other biarsenical probes, the conjugation reaction in three times molar excess of the peptide was rapid and required just 25 min for completion. SrtCrAsH-TC12 conjugate has a modest 6.6% increase of the absorption, compared to free probe; however, the fluorescence is increased by 390×, as commonly observed for biarsenical probes (Figure 2c,d). The excitation and emission maxima of the TC-free probe are 493 and 532 nm, respectively. The TC12-bound probe emits at 539 nm when excited at 512 nm (Figure S1). These values are similar to the 511 and 536 nm values of the parent CrAsH-TC12 complex.^33^ In order to determine the molar absorption coefficient of the SrtCrAsH-TC12 complex, a probe linker solution of known absorption was mixed with the TC12 peptide at various molar ratios from 0 to 5 to determine the first exact complex concentration. Then, it was converted to the molar coefficient reaching 50,770 M^–1^·cm^–1^ at pH 7.4 (Figure S2). It should be noted that although biarsenical probes contain arsenic(III), which is toxic and carcinogenic, the heavy metal is complexed either by EDT or TC motif, minimizing its side reactivity. There are numerous studies where this class of dyes was used to label proteins in live cells or insects without any issues related to target survival.^16^ That being said, potential users should be aware of necessary basic personal protection equipment and country-specific regulations of disposal of solutions containing arsenic.

### Preparation of Recognizable PTMs and Active Sortase A

With the SrtCrAsH-EDT_2_ probe in hand, we directed our attention to the preparation of post-translational modifications, which are represented here by small proteins: ubiquitin (Ub) and SUMO tag (small ubiquitin-like modifier). The attachment of a single ubiquitin molecule to a substrate regulates protein activity, cellular location, protein interactions, or endocytosis, with implications for various cellular processes and pathologies.^34,35^ SUMOylation regulates protein interactions, subcellular localization, transcriptional activity, and stability, playing critical roles in transcriptional regulation, response to stress, nuclear-cytosolic transport, and maintenance of genome integrity.^36,37^ We expressed and purified two proteins, Ub-LPNTG and SUMO-LPQTG, and then stored them at −80 °C. Their purity and mass correctness were confirmed using HPLC and ESI-MS (Figure S3 and Table S1). They contain the eSrtA recognition site (LPXTG) on their C terminus, thus allowing a transacylation reaction in which the C-terminal glycine is exchanged for a synthetic oligoglycine peptide located on the applied probe. It results in a transfer of the linker to the C terminus of ubiquitin or SUMO tag.

After the purification of chosen PTMs, we focused on the protein catalyst, which would allow their specific incorporation into the protein of interest (here GST-TC12, glutathione *S*-transferase with TC12 tag) via SrtCrAsH-EDT_2_ probe linker.^38^ eSrtA was expressed and purified in *Escherichia coli* and stored at −80 °C. Its purity and mass were confirmed using HPLC and ESI-MS (Figure S4, Table S1). Then, we examined enzyme activity using two different methods. First, we applied a fluorescence resonance energy transfer (FRET) substrate, which was designed to have two fluorescent molecules (a donor and an acceptor) attached to a peptide sequence specific to the enzyme of interest. Such a substrate is highly sensitive, capable of detecting minute changes in enzyme activity, and suitable for different types of enzymes and experimental conditions.^39,40^ For sortase A we chose a peptide with an aminobenzoyl group (Abz) and dinitrophenyl (DNP) group attached to a diaminopropionic acid (Dap) residue, Abz-YNLPETGA-Dap(DNP)-NH_2_. The nonfluorescent peptide cleaves into the fluorescent Abz-YNLPET-COOH (λ_ex_ = 320 nm, λ_em_ = 420 nm) product only when the enzyme is active (Figure S5).^39^ Because this test is based on substrate hydrolysis, sortase A activity has also been examined using model peptides containing recognition termini for their ligation, namely, YKNLPETGA and GGGKY. The ligation reaction was monitored by analytical high-performance liquid chromatography (HPLC) (Figure S5) and presented the formation of the correct product of the reaction, YKLPETGGGKY, confirming the sortase’s activity. Besides the desired ligation product, we also detected the YKLPET peptide. It is because of the reversibility of the reaction. The final product represents the LPETG motif that constitutes a substrate for sortase, which results in product hydrolysis. Therefore, the side product accompanies the sortagging reactions, and complete transformation of the substrate is practically impossible.^18^

### Production and Purification of GST-TC12 and HePTP-4C

The next step needed for the implementation of SrtCrAsH-EDT_2_ and tetracysteine chemistry was the expression and purification of the model target proteins. We have selected two model proteins. The first was glutathione *S*-transferase fusion from pGEX-6P1 possessing a C-terminal optimized tetracysteine motif (GST-TC12). We chose this protein as it is optimized for high-yield expression and ease of purification. Moreover, the crystal structure shows that the C terminus is exposed, minimizing the risk of steric hindrance during conjugation. The second target possessed two pairs of cysteines within its sequences. Both proteins were stable after the final transfer to a buffer optimal for eSrtA activity. However, it should be noted that HePTP-4C was stable only for a couple of days at 4 °C, and after some time, protein precipitation was observed, although not all protein was lost. To establish the mass of both proteins, we performed mass spectrometry experiments. Initial experiments on the electrospray ionization quadrupole time-of-flight (ESI-Q-TOF) instrument did not yield any results, even though we applied a denaturing mix of water, methanol, and formic acid (50:49:1). We therefore moved to the matrix assisted laser desorption ionization-time of flight (MALDI-TOF) instrument, which is better suited for high-mass protein complexes. The details are given in a separate section. Examination of both proteins in their purified state revealed a slightly larger mass than expected (Table S1). Since these proteins were measured using MALDI-TOF, this increase can be traced to adducts of matrix HCCA+K^+^ or its fragments. In order to facilitate the utilization of our linker, we deposited a plasmid with optimized TC12 (FLNCCPGCCMEP) that can be used to produce proteins with this motif in Addgene (plasmid 221852).

### One-Pot Sortase-Mediated Ubiquitination and SUMOylation of Proteins Using via SrtCrAsH-EDT_2_ Probe Linker

**Figure 3:**
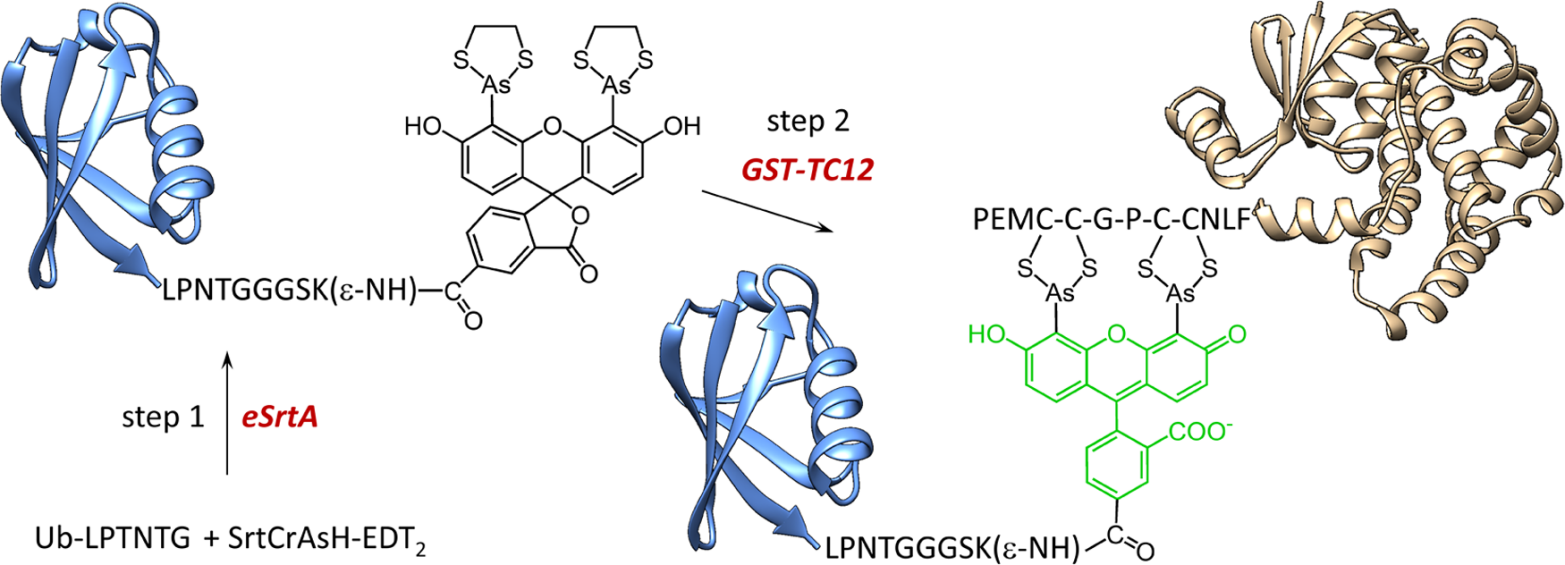
Concept representing the site-specific and fluorescently enhanced introduction of ubiquitin (PTM) to GST-TC12 using site SrtCrAsH-EDT_2_ as a linker probe. Step 1 represents sortase A (eSrtA)-mediated ubiquitination of the linker, while step 2 shows final conjugation to GST-TC12, highlighted by linker probe green fluorescence.

After the synthesis and purification of all required substrates and ligase, we concentrated on the one-pot site-specific conjugation. For that purpose, Ub-LPNTG was mixed first with a 2-fold excess of SrtCrAsH-EDT_2_ in the presence of sortase A to minimize hydrolysis as a side reaction. The nucleophile, represented by SrtCrAsH-EDT_2_, pushes the equilibrium of the reaction toward the product side (first step of Figure 3). An analogous reaction was run for SUMO-LPQTG and SrtCrAsH-EDT_2_ in the presence of sortase A. All sortase-mediated reactions were carried out for 2 h, and aliquots of those mixtures were saved for further SDS-PAGE and FPLC analysis. Then the sample was divided, and GST-TC12 was added at two molar ratios to reach a value of 1 and 3 relative to the biarsenical probe. We performed this test to establish how an excess of tetracysteine target influences the final efficiency of the reaction. Later analysis showed that the final efficiency of the modification reaction was not affected by this parameter (Figure S7). However, to push the reaction toward the final product and to avoid its decomposition, we recommend using a 1:3 molar ratio between the probe and target protein. Conjugation of the biarsenical probe to tetracysteine motif was carried out for 2 h (second step of Figure 3), as this time was sufficient for the completion of the reaction in both samples.

**Figure 4:**
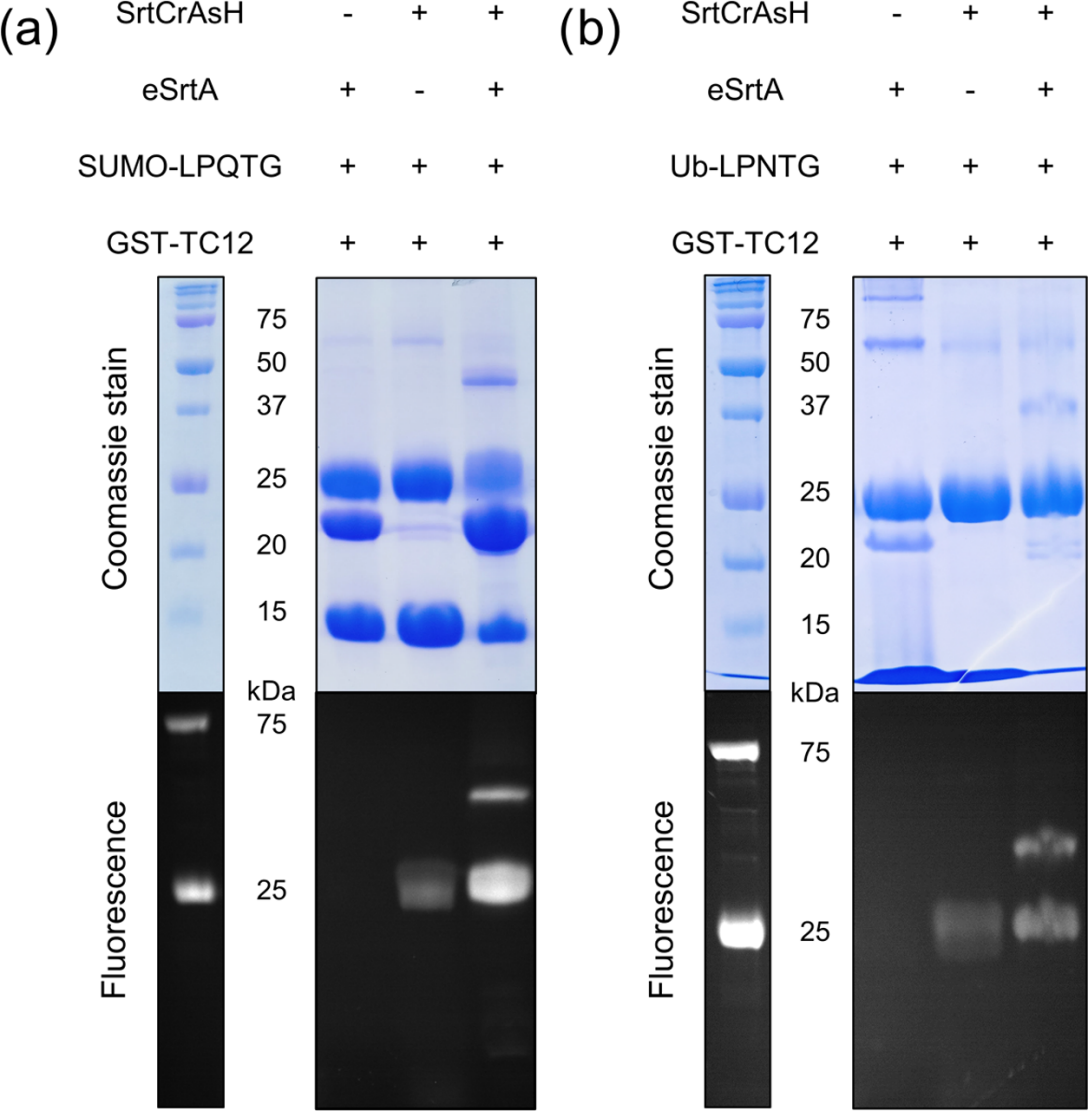
One-pot SUMOylation (a) or ubiquitination (b) of glutathione *S*-transferase (GST-TC12) via synthesized SrtCrAsH-EDT_2_ linker monitored by SDS-PAGE. Protein visualization by both Coomassie stain and fluorescence reveals that expected products SUMO-SrtCrAsH-GST-TC12 (a) and Ub-SrtCrAsH-GST- TC12 (b) are formed at around 37 kDa. In the controls without the SrtCrAsH linker or eSrtA sortase, no product is observed. The full images of the gels, additionally presenting standards and controls, are available in Supporting Information Figures S8 and S9.

In order to monitor reaction progress, samples were visualized using sodium dodecyl sulfate polyacrylamide gel electrophoresis (SDS-PAGE) in two different ways. First, gel was visualized in a gelbox equipped with 250 nm UV light to monitor the fluorescence intensity of particular bands, followed by staining with Coomassie in a standard protocol (Figure 4). As mentioned above, characteristic green fluorescence occurs when the biarsenical probe is attached to its tetracysteine motif. It remains quenched when capped with EDT due to rotation of arsenic atoms with bound EDT.^41^ Moreover, we and others have shown that proteins containing TC motifs or cysteine-rich environments remain conjugated with bound biarsenical probe in SDS gel and might be analyzed even at low concentrations.^42,43^ Reaction profiles demonstrated in Figure 4 show that expected final conjugates of SUMO-SrtCrAsH-GST-TC12 (Figure 4a) and Ub-SrtCrAsH-GST-TC12 (Figure 4b) are indeed formed (band at ∼37 kDa) in a one-spot reaction, however with different yield. About 6-fold less of the SUMO-SrtCrAsH-GST-TC12 product was observed compared to Ub-containing conjugate. No product is formed when either eSrtA sortase or SrtCrAsH linker is absent. The full images of the gels from Figure 4, additionally presenting standards and controls, are available in Supporting Information Figures S8 and S9.

**Figure 5:**
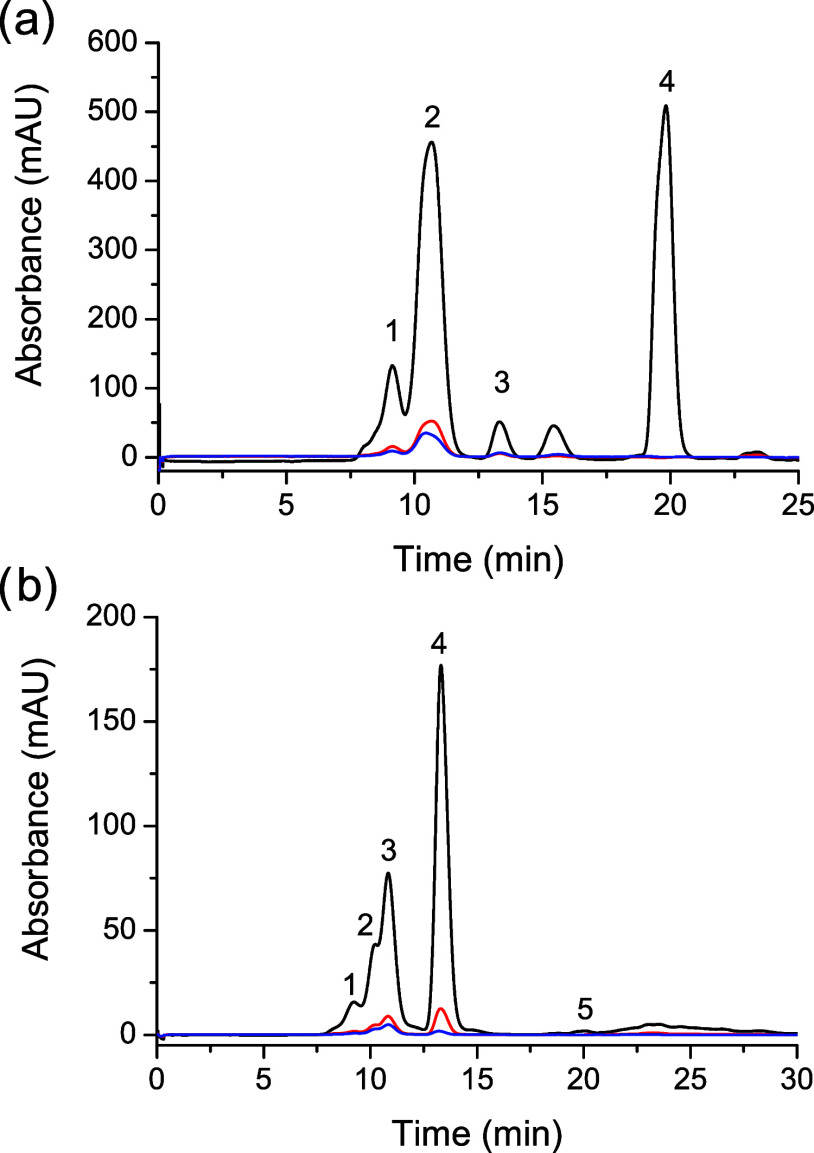
Size exclusion chromatography (SEC) for the Ub (a) and SUMO (b) ligation reactions with GST-TC12 and SrtCrAsH. (a) Obtained chromatogram presents several peaks: 1 refers to the mixture of a dimer of GST-TC12 with minimal SrtCrAsH labeling, 2 refers to the mixture of product Ub-SrtCrAsH-GST-TC12, labeled and unlabeled GST-TC12, 3 refers to the mixture of labeled and unlabeled ubiquitin, and 4 belongs to the GGSHHHHHH sequence, which formed in result of sortase cleavage between Thr and Gly residues of ubiquitin C terminus. (b) Chromatogram obtained for SUMO conjugate presents several peaks: 1 refers to the mixture of a dimer of GST-TC12 with minimal SrtCrAsH labeling, 2 refers to the SUMO-SrtCrAsH-GST-TC12 product, 3 refers to the mixture of SrtCrAsH labeled and unlabeled GST-TC12, 4 belongs to a mixture of the labeled and unlabeled SUMO, and 5 refers to the GGSHHHHHH sequence, which formed in result of sortase cleavage between Thr and Gly residues of ubiquitin C terminus. The reactions for Ub (a) and SUMO (b) were stopped and subjected to SEC on a Superdex 75 Increase 10/300 (Cytiva) in 50 mM Tris pH 7.5 and 150 mM NaCl. Black, red, and blue lines represent absorbances recorded at 220, 280, and 510 nm, respectively.

To provide additional proof of conjugation, we performed mass spectrometry experiments. Initially, the desalted reaction mixture was analyzed using the ESI-Q-TOF instrument. However, no signal was observed even when acidic conditions in the presence of methanol were added. We suspected that the final conjugate was not ionized efficiently. Therefore, we switched to MALDI-TOF. Utilizing this method, we were able to detect the desired product (Table S2). Besides the main product, SDS gels demonstrate bands belonging to SrtCrAsH-GST-TC12 as a side product at a mass of ∼28 kDa, which has also been detected by mass spectrometry. Finally, in order to monitor ligation reactions with more accuracy than SDS-PAGE, we analyzed reactions of the formation of Ub- and SUMO- conjugates with GST-TC12 using size exclusion chromatography (SEC). Figure 5 shows the chromatograms with observed signals at 220, 280, and 510 nm.

On the obtained chromatogram for the ubiquitin conjugate, we observed several peaks. The first one refers to the mixture of dimer of labeled and unlabeled GST, the second one to the mixture of product, labeled and unlabeled GST, the third one to the mixture of labeled and unlabeled ubiquitin, and the last one belongs to the GGSHHHHHH sequence, which formed as a result of sortase cleavage between Thr and Gly residues of ubiquitin C terminus. The chromatogram acquired for the SUMO conjugate presented a few peaks, as well. The first one belongs to the dimer of labeled and unlabeled GST, the second one belongs to the final product, the third one belongs to the labeled and unlabeled GST, the fourth one belongs to the labeled and unlabeled SUMO, and the last one belongs to the GGSHHHHHH sequence. Overall in both reactions, complete separation of the product was not possible because of the small difference in mass between it and the substrates. This, however, can be easily mitigated by including an affinity purification tag within the sequence of the PTM protein. Furthermore, the intensity of the peaks for SUMO and ubiquitin, as well as for the GGSHHHHHH sequence, together with previous results of SDS-PAGE analysis, suggest the substrate conversion for the ubiquitin is much higher than for SUMO. Therefore, in our further experiments with TC target proteins, we decided to proceed only with ubiquitin. Due to the nature of biarsenical probes, several considerations have to be made for long-time storage of SrtCrAsH protein conjugates. Most importantly, the samples cannot be stored in buffers containing dithiothreitol (DTT) or β-mercaptoethanol as both of those thiol compounds can in high concentrations compete for arsenic, leading to dissociation of the linker from TC12-containing protein. Instead, TCEP should be used to maintain the reduced state of the protein. Due to their fluorescent properties, SrtCrAsH conjugates should not be exposed to direct sunlight. Otherwise, the stability of the conjugate will be mostly dependent on the linked proteins.

### Conjugation of PTM to Sensitized HePTP via SrtCrAsH Probe Linker

**Figure 6:**
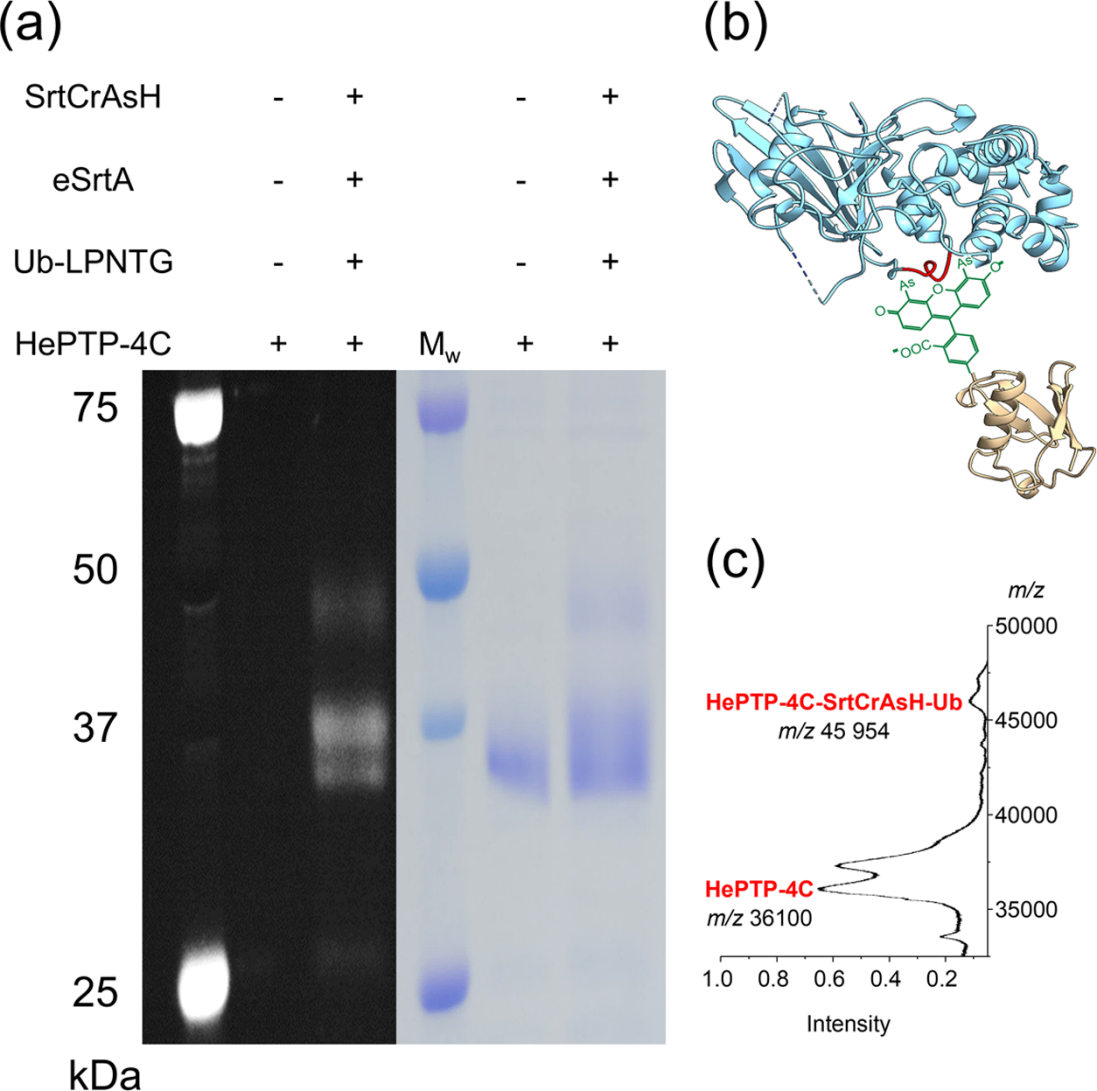
One-pot modification of HePTP with ubiquitin targeted to the inner sequence tetracysteine tag. (a) Lane 2 represents HePTP-4C, possessing an internal tetracysteine motif prior to modification. Lane 3 shows the product of the reaction with SrtCrAsH and ubiquitin. The gel was visualized in fluorescence and Coomassie stain (b). Graphical representation of the Ub-SrtCrAsH-HePTP-4C complex, highlighting the ability of the linker to the desired modification to the inner sequence of target protein. Mass spectrometry analysis of whole reaction (c) confirmed the presence of expected HePTP-4C-SrtCrAsH-ubiquitin with an expected mass of 45,954 Da. The fluorescent band with a mass of 36,100 kDa represents HePTP-4C labeled with an unmodified biarsenical probe linker that was added in excess over ubiquitin.

The addition of protein-based PTMs has been performed on multiple occasions by using various ligases. Two general approaches are utilized to incorporate the ligase recognition motifs in the POI. Either the termini of a target protein are modified with recognition sequences for the ligases (i.e., sortase), or the recognition motif is created by genetic code expansion.^44^ However, there are some issues with both approaches. The terminal modification with protein PTM is not natural, as both ubiquitin and SUMO modify the amino acid residues within the sequence of the protein.^45^ The second approach, related closer to nature, requires the introduction of unnatural amino acids, usually possessing a click-chemistry linker to attach ligase recognition motif. Unfortunately, the expansion of the genetic code is not always efficient, and depending on the protein and sites within a protein, it can vary between 10 and 100%.^13^ Our system can be used to introduce modifications within the sequence using a standard genetic code. It has the added advantage that the tetracysteine motif, thanks to its small size, can be placed within the sequence of the target protein. To this end, we utilized a previously characterized mutant of hematopoietic protein tyrosine phosphatase (HePTP) that has a tetracysteine tag located in the center of the protein.^46^ Following our optimized procedure, we could attach ubiquitin to our target protein (Figure 6). The reaction was successfully completed, although the efficiency was notably lower than with the model proteins shown above. However, GST possess the optimized 12 amino acids long TC-tag at the terminus, which makes it more available for modification and enhances TC-tag labeling. HePTP was modified with a minimal two pairs of cysteine residues to avoid disturbing the protein folding.^46^

### DeSUMOylation of SUMO-SrtCrAsH-GST Conjugate

In the final stage of our study, we carried out the experiment of removing the SUMO tag from the final SUMO conjugate by ubiquitin-like protease (ULP1), which specifically cleaves the isopeptide bond formed between the C-terminal glycine of the SUMO tag and the lysine residue of the protein.^47^ ULP1 plays a pivotal role in the maturation of SUMO precursors and the deconjugation of SUMO from target proteins. For the deSUMOylation purpose, to the reaction mixture, we added ULP1 protease and left it for 16 h at 24 °C. The sample was analyzed by SDS-PAGE with visualization by fluorescent gel scanning and Coomassie staining (Figure S10). As a result, the band that refers to the final product disappeared after incubation, showing that biologically relevant cleavage of the SUMO-SrtCrAsH-GST-TC12 conjugate is possible.

## Conclusions

In summary, we developed a one-pot strategy for the introduction of various post-translational modifications at selected positions of the protein utilizing the benefits of sortase-mediated ligation and biarsenical fluorescent linkers, such as high selectivity, sensitivity, versatility, and compatibility with advanced imaging techniques. This strategy streamlines the protein modification process because it eliminates the need to isolate and purify intermediate products and reduces the reaction time and overall complexity. The PTMs are precisely incorporated in some loops or secondary structure elements, and such flexibility caters to various experimental or therapeutic needs. PTMs, such as ubiquitination or SUMOylation, can significantly alter a protein’s function, stability, interaction partners, and localization. By tagging these modified proteins with fluorescent probes, proteins’ physical properties and cellular roles can be directly correlated under different physiological conditions or in disease states. Therefore, the presented methodology and our developed linker SrtCrAsH-EDT_2_ for one-pot labeling could be employed to investigate the impact of PTMs on proteins, influencing their structure, function, localization, and interactions within the cellular context. Understanding their impact is essential for unraveling the intricate mechanisms that govern cellular function and dysfunction.

## Experimental Procedures

### Materials

1,2-Ethanedithiol (EDT), triisopropylsilane (TIPS), 2-[(2-hydroxy-1,1-bis(hydroxymethyl)ethyl)amino]ethanesulfonic acid (TES), ethylenedinitrilotetraacetic acid (EDTA), β-mercaptoethanol, CaCl_2_·2H_2_O, HgO, AsCl_3_, 5-carboxyfluorescein, *N*,*N*′-diisopropylcarbodiimide (DIC), acetonitrile (MeCN), HCl (trace-metal grade), hydrazine monohydrate, imidazole, ammonium carbonate, and sodium chloride were purchased from Merck Millipore. *N*,*N*-Dimethylformamide (DMF) and HCl were from VWR Chemicals. Diethyl ether, chloroform, and dimethyl sulfoxide (DMSO) were from Avantor Performance Materials Poland (Gliwice, Poland). Tris(2-carboxyethyl)phosphine hydrochloride (TCEP), Oxyma Pure, trifluoroacetic acid (TFA), *N*,*N*-diisopropylethylamine (DIEA), piperidine, *N*-methyl-2-pyrrolidone (NMP) TentaGel S Ram, TentaGel S PHB resin, and Fmoc-protected amino acids were obtained from Iris Biotech GmbH (Marktredwitz, Germany). Tryptone, yeast extract, LB Broth, agar, agarose, isopropyl-β-d-1-thiogalactopyranoside (IPTG), and sodium dodecyl sulfate (SDS) were purchased from Lab Empire. Ampicillin, chloramphenicol, 1,4-dithiothreitol (DTT), and Tris base were from Roth. 4-(2-hydroxyethyl)piperazine-1-ethanesulfonic acid sodium salt (HEPES) was from Bioshop. Abz-YNLPETGA-Dap(DNP)-NH_2_ was ordered from ProteoGenics. Aqueous solutions were configured with Milli-Q water (18.2 MΩ·cm^–1^, 0.22 μm filter). Microporous membrane filters (0.22 and 0.45 μm) were used for further purification (Jet Biofil, China). All of the reagents were purchased from commercial suppliers and used accordingly. All buffers were prepared with Milli-Q water obtained with a deionizing water system (Merck KGaA).

### Peptide Synthesis

All peptides were synthesized by utilizing solid-phase synthesis on TentaGel S PHB resin (substitution 0.22 mmol/g) using the Fmoc strategy and a Liberty Blue 2.0 microwave-assisted synthesizer (CEM). The reagent excess, cleavage, and purification were performed as previously described.^48,49^ The Dde protection group was removed by 5% (w/v) solution of hydrazine monohydrate in DMF (25 mL/g of peptide-resin) three times for 3 min at room temperature. Coupling of lysine ε-NH_2_ 5-carboxyfluorescein (6 molar equiv) was performed with Oxyma Pure (3 molar equiv) and DIC (3 molar equiv). Peptides were cleaved from the resin with a mixture of TFA/TIPS/H_2_O (95:2.5:2.5 v/v/v) over a period of 2 h, followed by precipitation in cold (−70 °C) diethyl ether. The crude peptide was collected by centrifugation, dried, and purified via RP-HPLC (Varian ProStar) on a Varian Pursuit XRs C18, 250·21.2 mm, 5 μm, 100 Å PREP HPLC column, using a gradient of MeCN in 0.1% TFA/water from 1 to 70% over 40 min at a flow rate of 10.0 mL/min. Fractions containing pure product GGGSK(ε-5-FAM) were collected, identified by ESI mass API 2000 Applied Biosystems spectrometry, and lyophilized. The list of synthesized peptides, along with their calculated and observed mass is presented in Table S1. Concentrations of peptides were determined by NanoDrop A280.

### Synthesis of SrtCrAsH Probe Linker

The synthesis followed the standard route for biarsenical probes as described previously.^15,16^ To 14 mg of pure GGGSK(ε-CrAsH) peptide, 8.5 mg of HgO was added along with 300 μL of TFA. The mixture was incubated at room temperature for 2 h with shaking. After that, TFA was evaporated with a nitrogen stream. After that, 750 μL of water was added, the mixture was frozen at −80 °C, and freeze-dried. This procedure was repeated twice. To 6.2 mg dried mercuric derivative, 37 μL of dried NMP was added along with 6 μL of AsCl_3_, 5 μL of DIEA, and 7 μg of Pd(OAc)_2_. The mixture was stirred at 60 °C for 3 h. Next 370 μL of 0.1 M potassium phosphate buffer pH 7.0: acetone mixture (1:1 volume) was added along with 6 μL of EDT. The reaction was stirred for 15 min, and then the mixture was extracted with 200 μL of chloroform twice. The aqueous phase was then diluted 1:1 with 10 mM (NH_4_)_2_CO_3_ and purified on HPLC (Waters 1525) utilizing a gradient of MeCN in 10 mM (NH_4_)_2_CO_3_ on Phenomenex C18 preparative column, Gemini, 5 μm, 10·250 mm. Fractions containing the SrtCrAsH-EDT_2_ were pooled, divided into Eppendorf tubes, and dried using a SpeedVac. The probe linker was stored at −20 °C.

### Expression of Sortase A (eSrtA)

*S. aureus* sortase A pentamutant (eSrtA) in pET29 was a gift from David Liu (Addgene plasmid # 75144).^50^ The eSrtA plasmid was transformed into BL21 (DE3) RIL *E. coli* competent cells for protein expression. The eSrtA was expressed and purified using previously described protocols with slight modifications.^51,52^ Briefly, the primary culture was grown overnight at 37 °C in an LB medium containing 50 μg/mL kanamycin. The secondary culture was inoculated in 1L of LB medium by adding 1% (v/v) primary culture and was grown at 37 °C until A_600_ reached 0.4–0.6. Protein expression was induced with 0.2 mM IPTG for 3 h at 37 °C. The cells were harvested by centrifugation (4000 rpm, 15 min, and 4 °C), resuspended in resuspension buffer (10 mM Tris pH 7, 40 mM NaCl, and 10 mM imidazole), and lysed by sonication, and the cell lysate was clarified by centrifugation (10,000 rpm, 30 min, and 4 °C). The eSrtA was purified by Ni^2+^-NTA affinity purification. The supernatant was incubated with Ni^2+^-NTA beads pre-equilibrated in resuspension buffer at 4 °C for 2 h. The beads were subsequently washed with wash buffer (10 mM Tris pH 7, 500 mM NaCl, and 30 mM imidazole). The protein was eluted with elution buffer (50 mM Tris pH 7.4, 150 mM NaCl, and 350 mM imidazole), concentrated in an Amicon-Ultra concentrator (3/10 kDa MWCO), and desalted on a PD-10 column in desalting buffer (50 mM Tris 7.4 and 150 mM NaCl). The purity of each of the purified protein samples was analyzed by SDS-PAGE and RP-HPLC. Molecular masses of the proteins were confirmed by ESI-MS (Table S1).

### Fluorometric Activity Assay for eSrtA Using Abz-YNLPETGA-Dap(DNP)-NH_2_

The fluorometric activity experiment was performed in 1 mL of a reaction mixture containing buffer (50 mM Tris pH 7.5, 150 mM NaCl, 10 mM CaCl_2_, and 2 mM β-mercaptoethanol), eSrtA (25 μM), and 25 mM Abz-YNLPETGA-Dap(DNP)-NH_2_. Reactions were initiated by the addition of the enzyme and were monitored by measuring the fluorescence spectra from 400 to 600 nm and observing the increase in fluorescence (λ_ex_ = 320 nm, λ_em_ = 420 nm, 37 °C) at different time points.^40^

### HPLC-Monitored Model Peptide Activity Assay for eSrtA

The assay was carried out in 20 μL of reaction mixture containing buffer (50 mM Tris at pH 7.5, 150 mM NaCl, 10 mM CaCl_2_, and 2 mM β-mercaptoethanol), eSrtA (25 μM), 0.5 mM N-terminal substrate (YKNLPETGA), and 1 mM C-terminal substrate (GGGKY). Reactions were initiated by the addition of enzyme and incubated at 37 °C for 2 h. The reaction was quenched by the addition of 80 μL of 0.1% TFA, and the reaction mixtures (final volume, 100 μL) were injected directly into a C18 analytical RP-HPLC column. The reaction was analyzed using a MeCN/0.1% TFA gradient from 5 to 35% in 40 min and from 35 to 85% in 20 min, and absorbance was recorded at 220 nm. To confirm the composition and identity of each product, the peaks were collected and analyzed by ESI-MS (Table S1).

### Expression of Ubiquitin and SUMO with eSrtA Recognition Motif (LPxTG) at Their C Terminus

pETM11-SUMO-SNCA-GFP was a gift from Dmytro Yushchenko (Addgene plasmid # 107292).^53^ Plasmid encoding Ubiquitin WT was a gift from Rachel Klevit (Addgene plasmid # 12647).^54^ Both plasmids were used as templates for PCR amplification of SUMO-2 and ubiquitin, respectively, using these specific primers (listed below). Two reverse primers were used for incorporating LPQTG at the C-term of human SUMO-2. The first primer, which had LPQT, was used for SUMO-2 gene amplification. This amplified gene was used as a template amplifying the gene encoding human SUMO-2 with LPQTG and the *Bam*HI restriction enzyme site at its 3′-end. Ub: Fwd 5′- AAAAAACATATGCAGATCTTCGTCAAGACGTTAACC; Ub-LP Rev 5′- AAAAAAGGATCCACCGGTGTTCGGTAGTCTTAAGACAAGATG; SUMO Fwd 5′ GGGGGGCATATGGGCAACGATCACATTAACCTGAAAG; SUMO Rev 1 5′ – AAAAAACGGTCTGCGGCAGGAACACGTCAATGGTG; SUMO Rev 2 5′ – AAAAAAGGATCCACCGGTCTGCGGCAGGAAC. The obtained amplicons were digested with NdeI and *Bam*HI restriction enzymes and subcloned into the eSrtA(4S-9) pET29b vector (Addgene #75146) under the same sites.^55^ The plasmids containing Ub-LPNTG and SUMO-LPQTG were sequenced to confirm the presence of insertion and deposited in Addgene under accession numbers #221850 and #221851. Next, they were transformed into *E. coli* BL21(DE3) RIL competent cells for protein expression. The primary culture was grown overnight at 37 °C in LB medium containing 50 μg/mL kanamycin. The overnight culture was used for the inoculation of the secondary LB medium culture, which was grown at 37 °C until A_600_ reached 0.4–0.6. This was followed by induction with 0.2 mM IPTG for protein expression for 3 h at 37 °C (Ub-LPNTG and SUMO-LPQTG). Both proteins were purified similarly to eSrtA. After elution, both proteins were concentrated in an Amicon-Ultra concentrator (3 kDa MWCO) and desalted on a PD-10 column using desalting buffer (50 mM Tris 7.4 and 150 mM NaCl). The purity of each of the purified protein samples was analyzed using SDS-PAGE and RP-HPLC. Molecular masses of the proteins were confirmed by ESI-Q-TOF MS (Compact, Bruker) (Table S1).

### Expression of Protein Targets

The plasmid encoding HePTP containing a tetracysteine motif at position 211 and His-tag was a gift from Prof. Anthony Bishop.^46^ The protein was prepared as previously described.^15^ Briefly, the *E. coli* BL21(DE3) RIL transformed with pBAD-HePTP-4C@211 vector were cultured in LB medium. Incubation at 37 °C was conducted until the OD600 reached 0.8. After that, arabinose was added to a final concentration of 0.04%, and the temperature was decreased to 26 °C overnight. The protein was purified using Ni^2+^-NTA following the manufacturer’s protocol. The GST-TC12 protein was expressed by using a modified pGEX6P1 plasmid. Plasmid mutagenesis is described in the Supporting Information. Plasmid was used to transform the *E. coli* BL21(DE3) RIL strain using the heat shock method. The cells were grown at 37 °C in LB medium until OD600 reached 0.8. After that, IPTG was added to the final concentration of 0.1 mM, and incubation was continued for 4 h at 30 °C. The pelleted bacteria were frozen. Cells were resuspended in 20 mM Tris pH 7.45 with 150 mM NaCl and 1 mM TCEP and sonicated. The lysate was centrifuged at 16,000*g* at 4 °C, and the supernatant was applied to agarose-GSH resin. Incubation was performed for 2 h at 4 °C. After that, the resin was washed four times with 50 mL of 20 mM Tris pH 7.45 with 150 mM NaCl and 1 mM TCEP. Elution was effected 20 mM Tris pH 7.45 with 150 mM NaCl 1 mM TCEP and 20 mM GSH. Pooled fractions were concentrated on an Amicon-Ultra concentrator (10 kDa MWCO). Next, proteins were purified on the ENrich 70 SEC column on FPLC (NGC, Bio-Rad). The pure protein fractions were pooled and concentrated on the Amicon-Ultra concentrator (10 kDa MWCO). The purity of each of the purified protein samples was analyzed by SDS-PAGE. Molecular masses of the proteins were confirmed by ESI-Q-TOF MS (Compact, Bruker; Table S1).

### Sortase-Mediated Ubiquitination and SUMOylation of Proteins Using SrtCrAsH-EDT_2_

The conjugation reaction was carried out by adding 150 μM probe linker to 75 μM ubiquitin, 75 μM SUMO tag, and 25 μM eSrtA in reaction buffer (50 mM Tris pH 7.5, 150 mM NaCl, 10 mM CaCl_2_, and 2 mM TCEP) with agitation at 37 °C for 2 h. Then, the target TC proteins were added at molar ratios 1:1 and 3:1 relative to the probe, and the reactions were carried out for 2 h at 24 °C with agitation. Reactions were analyzed by SDS-PAGE with visualization by Coomassie staining and fluorescent gel scanning, and on SEC-70 gel filtration column (Bio-Rad) equilibrated with 50 mM Tris pH 7.5 and 150 mM NaCl.

### MALDI-TOF Measurements

Before the analysis by matrix-assisted laser desorption/ionization time-of-flight mass spectrometry (MALDI-TOF MS) micropurification and concentration of protein samples were performed using in-house produced reversed-phase C18 StageTips. StageTip for each sample was wet with acetonitrile, stabilized with water, and loaded with the sample.^56^ The loaded sample was purified with water and then eluted with 60% acetonitrile in water (2 μL followed by 4 μL). One μL portion of each sample was spotted on the polished steel MALDI target plate, dried, and covered with HCCA matrix (α-cyano-4-hydroxycinnamic acid, 10 mg/mL 60% acetonitrile in water or 60% acetonitrile/0.1% trifluoroacetic acid in water). All solutions were prepared using LC-MS grade reagents and MiliQ water. Dried samples were analyzed by using the MALDI-TOF ultrafleXtreme instrument (Bruker). The spectra were obtained in a linear positive ion mode in the mass range of 5–60 kDa. Protein Standard II (Bruker) was used for calibration purposes.

### Expression of ULP1

pFGET19_Ulp1 plasmid encoding yeast ULP-1 SUMO protease was a gift from Hideo Iwai (Addgene plasmid # 64697). The protein was expressed and purified as previously described.^57^

### DeSUMOylation Assay

ULP-1 as a SUMO protease was activated by preincubating it with 10 mM dithiothreitol (DTT) prior to the deSUMOylation reaction. 10 μM of the reaction mixture was later incubated with 1 μM ULP-1 in a buffer containing 150 mM NaCl, 50 mM Tris pH 7.5, and 5 mM β-mercaptoethanol at 37 °C for 16 h. The reaction mixture was analyzed by SDS-PAGE with visualization by fluorescent gel scanning and Coomassie staining.

### Size Exclusion Chromatography (SEC)

This method was applied for the analysis and collection of the modified target proteins. The reactions were quenched and subjected to SEC on a Superdex 75 Increase 10/300 (Cytiva) in 50 mM Tris at pH 7.5 and 150 mM NaCl. Absorbance was recorded at 220, 280, and 510 nm. The peaks were collected and analyzed by an API 2000 Applied Biosystems electrospray ionization (ESI) mass spectrometer.

## Supporting Information Available

The experimental data set used in this study is publicly available via the Zenodo repository at DOI: 10.5281/zenodo.14003996. The Supporting Information is available free of charge at https://pubs.acs.org/doi/10.1021/acsomega.4c05828.

Additional experimental details and characterization data for peptide derivatives and protein conjugates (PDF)
