# Supplementary material for: Site-Specific and Fluorescently Enhanced Installation of Post-Translational Protein Modifications via Bifunctional Biarsenical Linker
Source: ACS Omega. 2024 Oct 30;9(45):45127–37. doi: 10.1021/acsomega.4c05828 (PMC11561763; doi:10.1021/acsomega.4c05828)
Supplement: Supplementary file 1 — ao4c05828_si_001.pdf [file ao4c05828_si_001.pdf]

Supporting information

Site-specific and fluorescently-enhanced installation of post-translational protein modifications via bifunctional biarsenical linker

Anastasiia Antonenko[§1], Adam Pomorski[§1], Avinash Kumar Singh[1,3], Katarzyna Kapczyńska[2], Artur Krężel*[1]

[1]*Department of Chemical Biology, Faculty of Biotechnology, University of Wroclaw, Joliot-Curie 14a, 50-383 Wrocław, Poland*

[2]*Department of Immunology of Infectious Diseases, Hirszfeld Institute of Immunology and Experimental Therapy, Polish Academy of Sciences, Wrocław, Poland*

[3]*Department of Laboratory Medicine and Pathology, Mayo Clinic, Rochester 55901, MN, USA*

[§] these authors contributed equally

*corresponding author e-mail: Artur Krężel, artur.krezel@uwr.edu.pl

Keywords: CrAsH, PPT, protein engineering, sortase, SUMO, ubiquitin

Additional methods

Insertion of TC12 into GST. Third-generation tetracycline sequence FLNCCPGCCMEP [1] was inserted on the C-terminus of monomeric glutathione S-transferase located on pGEX6P1 plasmid using a PCR with quick-change mutagenesis protocol. Due to the length of the insertion, the reaction was performed in two steps using primer pairs described below:

1F: 5’ CCT CCA AAA TCG GAT TTC TTG AAT TGT TGC CCA GGA CTG GAA TGT TCC 3’

1R: 5’ GGA ACA GAA CTT CCA GTC CTG GGC AAC AAT TCA AGA AAT CCG ATT TTG GAG G 3’

2F: 5’ GAA TTG TTG CCC AGG ATG CTG TAT GGA ACC ATA GCT GGA AGT TCT GTT CC 3’

2R: 5’ GGA ACA GAA CTT CCA GCT ATG GTT CCA TAC AGC ATC CTG GGC AAC AAT TC 3’

The ready construct was used to transform *E. coli* DH10 cells using heat shock. The cells were cultured overnight in LB and collected. The plasmid DNA was extracted using the GeneJet plasmid miniprep kit (Thermo Scientific), following the manufacturer’s protocol. The mutagenesis was confirmed with sequencing.

Characterization of the SrtCrAsH linker molecule. All cuvette based fluorescence measurements were performed in Fluoromax-4 spectrofluorimeter (HoribaYvon Jobin) equipped with Peltier module for temperature control and mixing. All measurements were performed at 25 °C. Molar absorption coefficient of the biarsenical probes used in this study was established by titration with TC12 peptide. Biarsenical probes with estimated final concentration of 1 μM, based on average molar absorption coefficient of 50 000 $M^{-1} \times cm^{-1}$, were added to solution with 50 mM Hepes pH 7.4, 150 mM NaCl and 1 mM TCEP. Next absorbance spectra in the range of 425-575 nm was measured using V-650 spectrophotometer (Jasco), together with 3D excitation/emission spectra. The solution was aliquoted into Eppendorf tubes and TC12 peptide was added to the final concentration ranging from 50 nM to 25 μM. The samples were incubated for 3 h in room temperature with stirring in darkness.

Sample with the highest concentration of peptide was used to measure 3D excitation/emission spectra of the conjugate. For the rest of measurements excitation was set to 512 nm and emission collected at 539 nm. The inflection point in the graph of emission vs. concentration of TC12 denoted the true concentration of SrtCrAsH and hence molar absorption coefficient was calculated. The kinetic of fluorescence increase upon conjugation of SrtCrAsH to TC12 was measured using the same buffer, excitation and emission parameters. The concentration of SrtCrAsH was 1 μM, whereas TC12 was 3 μM.

Table S1. A list of calculated and experimental average molecular masses of peptides and proteins synthesized in this study. Except where indicated, peptides and proteins were measured on Compact Q-TOF (Bruker).

Peptide/protein/probe	Mass average calculated (Da)	Mass average experimental (Da)
GGGSK	404.4	404.8
GGGSK(ε-5-FAM)	761.2	761.2
SrtCrAsH-EDT_2	1,094.3	1,094.1
NH_2-FLNCCPGCCMEP-NH_2 (TC12)	1,315.6	1,315.2
YKNLPETGA	992.1	992.5
GGGKY	480.5	480.7
Ub-LPNTG	9,630.9	9,631.4
SUMO-LPQTG	10,240.5	10,241.6
eSrtA	17,854.1	17,852.2
GST-TC12*	27,000	27,412
HePTP-4C*	35,855	36,100

*Measured on MALDI-TOF

Ub-LPNTG amino acid sequence:

MQIFVKTLTGKTITLEVEPSDTIENVKAKIQDKEGIPPDQQRLIFAGKQLEDGRTLSDYNIQ
KESTLHLVLRLPNTGGSHHHHHH

SUMO-LPQTG amino acid sequence:

MGNDHINLKVAGQDGSVVQFKIKRHTPLSKLMKAYCERQGLSMRQIRFRFDGQPINE
TDTPAQLEMEDEDTIDVFLPQTGGSHHHHHH

eSrtA amino acid sequence:

MQAKPQIPKDKSKVAGYIEIPDADIKEPVYPGPATREQLNRGVSFAEENESLDDQNISIAGHTFIDRP
NYQFTNLKAAKKGSMVYFKVGNETRKYKMTSIRNVKPTAVEVLDEQKGKDKQLTLITCDDYNEETGVW
ETRKIFVATEVKLEHHHHHH

GST-TC12 amino acid sequence:

MSPILGYWKIKGLVQPTRLLLEYLEEKYEEHLYERDEGDKWRNKKFELGLEFPNLPYYIDGD
VKLTQSMAIIRYIADKHNMLGGCPKERAEISMLEGAVLDIRYGVSRIAYSKDFETLKVDFLS
KLPEMLKMFEDRLCHKTYLNGDHVTHPDFMLYDALDVVLYMDPMCLDAFPKLVCFKKRIEAI
PQIDKYLKSSKYIAWPLQGWQATFGGGDHPPKSDFLNCCPGCCMEP

HePTP-4C amino acid sequence:

MGSDKIHHHHHHMNTPREVTLHFLRTAGHPLTRWALQRQPPSPKQLEEEFLKIPSNFVSPED
LDIPGHASKDRYKTILPNPQSRVCLGRAQSQEDGDYINANYIRGYDGKEKVYIATQGPMPNT
VSDFWEMVWQEEVSLIVMLTQLREGKEKCVHYWPTEEETYGPFQIRIQDMKECPEYTVRQLT
IQYQEERRSVKHILFSAWPDHQTPCCPGCCESAGPLLRLVAEVEESPETAAHPGPIVVHCSA
GIGRTGCFIATRIGCQQLKARGEVDILGIVCQLRLDRGGMIQTAEQYQFLHHTLALYAGQLP
EEPSP

Table S2. A list of calculated and experimental average molecular masses of peptides and proteins synthesized in this study.

Conjugate	Mass average calculated (Da)	Mass average experimental (Da)
Ub*-LPNTG-SrtCrAsH-GST	36,643	36,915
SUMO*-LPQTG-SrtCrAsH-GST	37,386	37,392
Ub*-LPNTG-SrtCrAsH-HePTP-4C	45,601	45,954

*The eSrtA removes the C-terminal fragment (GGSHHHHHH) of the PTM proteins. Therefore, the expected sequence after conjugation to SrtCrAsH is the following (bolded and underlined parts represent the rest of the conjugate):

Ub-LPNTG amino acid sequence:

MQIFVKTLTGKTITLEVEPSDTIENVKAKIQDKEGIPPDQQRLIFAGKQLEDGRTLSDYNIQKESTLHLVLRLPNT**GGGSK(CrAsH)-GST-TC12** or **GGGSK(CrAsH)-HePTP-4C**

SUMO-LPQTG amino acid sequence:

MGNDHINLKVAGQDGSVVQFKIKRHTPLSKLMKAYCERQGLSMRQIRFRFDGQPINETDTPAQLEMEDEDTIDVFLPQT**GGGSK(CrAsH)-GST-TC12**

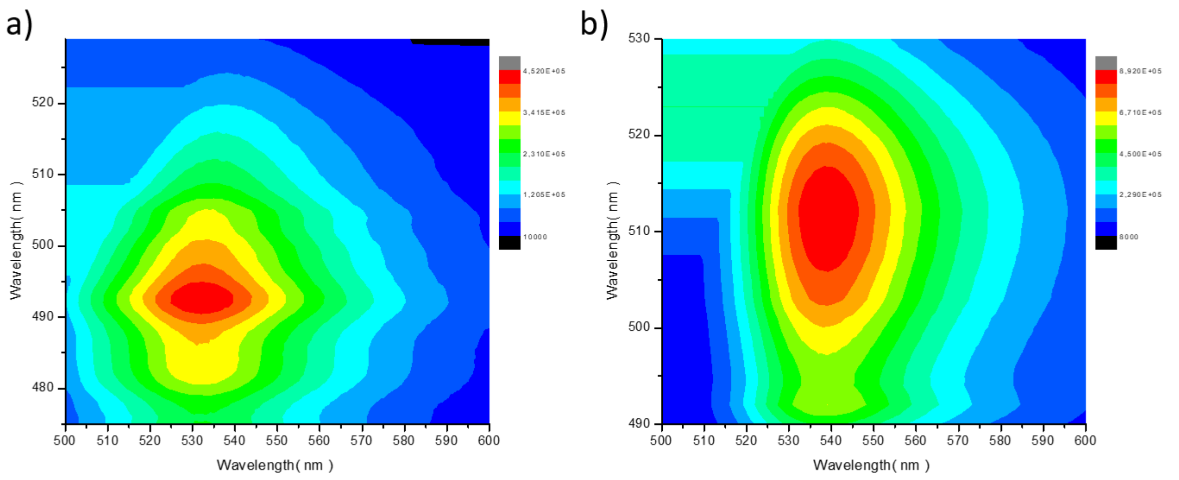

Figure S1. 3D fluorescence spectra. (a) Free probe maximum $\lambda_{ex}/\lambda_{em}$ is 493/532 nm. Due to quenched fluorescence, the instrument was set up for higher sensitivity than with TC12. (b) For the TC12 complex the maximum of $\lambda_{ex}/\lambda_{em}$ is 512/539 nm.

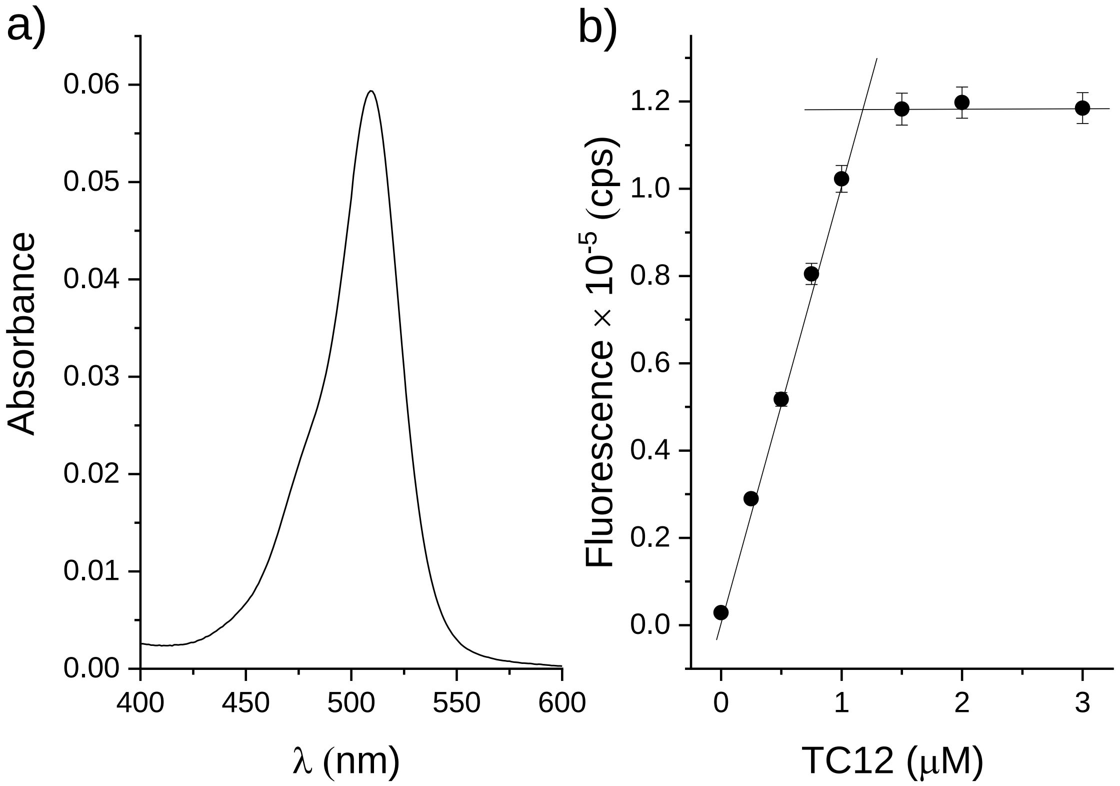

Figure S2. Determination of molar absorption coefficient of SrtCrAsH-EDT$_2$. (a) A solution of the linker probe was made and its absorbance was measured. (b) Simultaneously, it was aliquoted, and different molar ratios of TC12 peptide were added to precisely determine the concentration assuming the expected 1:1 binding. The calculated value of the molar extinction coefficient of SrtCrAsH-EDT$_2$ is 50,770 ± 1,000.

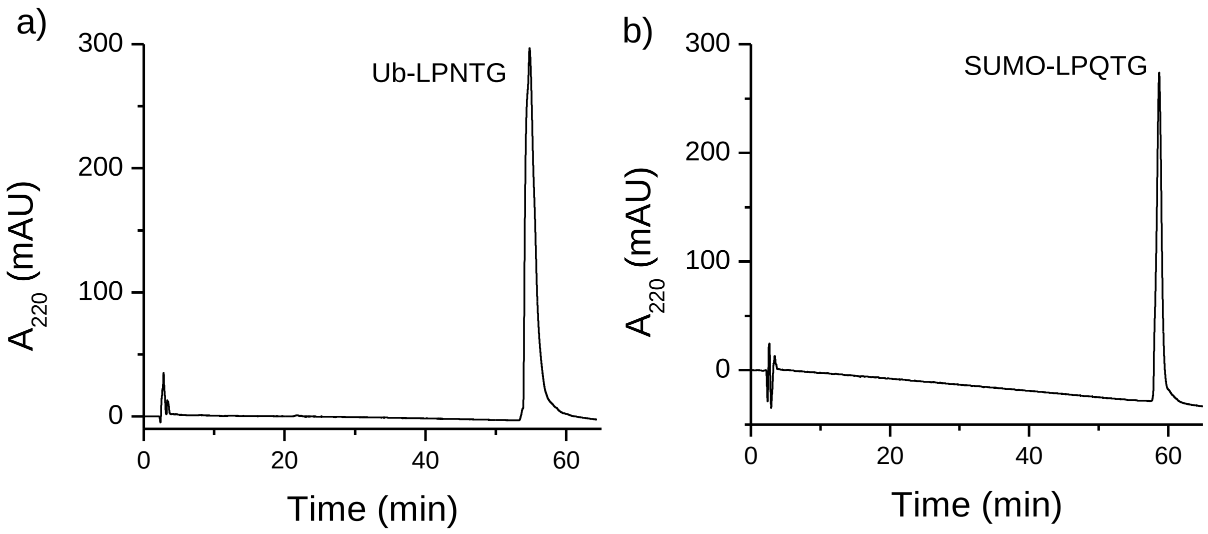

Figure S3. C18 RP-HPLC analysis of expressed PTMs: Ub-LPNTG (a) and SUMO-LPQTG (b). Proteins were eluted by applying the same gradient: from 5 to 35% in 40 min and from 35 to 85% MeCN/0.1% TFA in 20 min at a 1 mL/min flow rate. Absorbance was recorded at 220 nm. Peak fractions were collected, and their identities were analyzed by ESI-MS (**Table S1**).

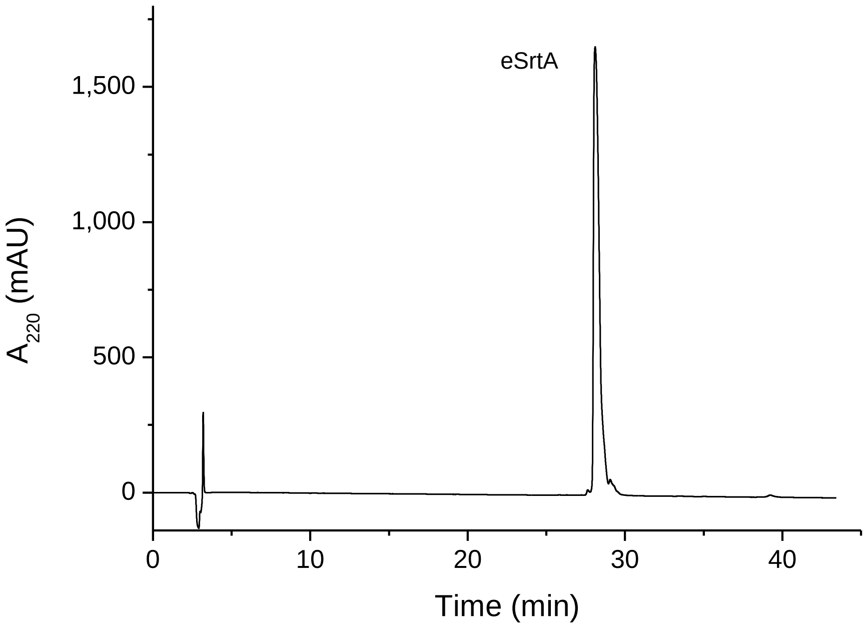

Figure S4. C18 RP-HPLC analysis of eSrtA. It was eluted by applying a gradient from 5 to 35% in 40 min and from 35 to 85% MeCN/0.1% TFA in 20 min at a 1 mL/min flow rate. Absorbance was recorded at 220 nm. Peak fraction was collected, and its identity was confirmed by ESI-MS.

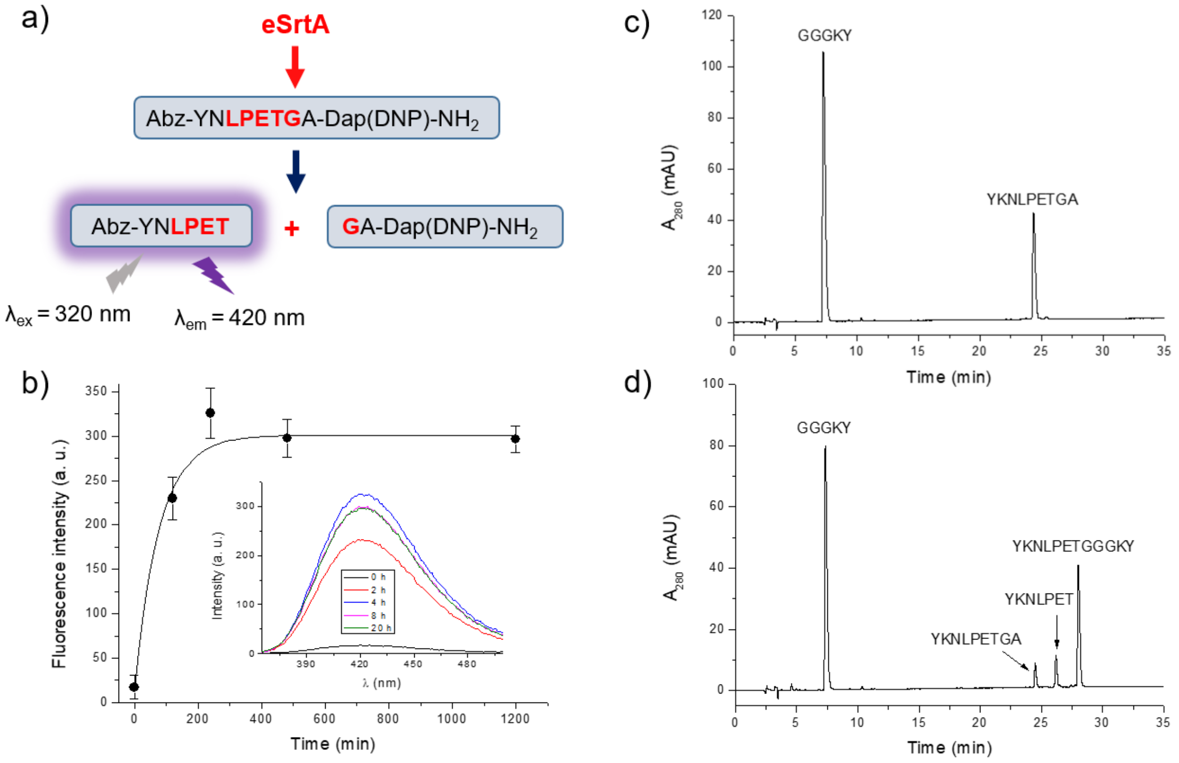

Figure S5. Fluorometric and HPLC activity assays for the eSrtA were used in this study. (a) Sortase activity was carried out using the specific FRET substrate Abz-YNLPETGA-Dap(DNP)-NH_2. When hydrolyzed (reverse to the ligation reaction), characteristic fluorescence of Abz-YNLPET at 420 nm was measured upon excitation at 320 nm. (b) Reaction kinetics of 0.5 mM substrate hydrolysis was monitored for 20 h using the presence of 25 µM eSrtA at 37 °C. Inset indicates emission spectra at 0-24 h time points, a.u. denotes arbitrary units. (c) HPLC profile of model peptides mixture: 0.25 mM N-terminal YKNLPETGA and 0.5 mM C-terminal GGGKY. (d) HPLC profile for the ligation reaction of model peptides incubated with 25 µM eSrtA at 37°C after 2 h. YKNLPETGGGKY is the desired product, and YKNLPET is the hydrolysis product. Reactants were separated on a C18 column (250 mm × 4.6 mm, 5.0 μm) in the gradient of 5−35% of MeCN in 40 min and 35−85% of MeCN in the next 20 min, and absorbance was recorded at 280 nm.

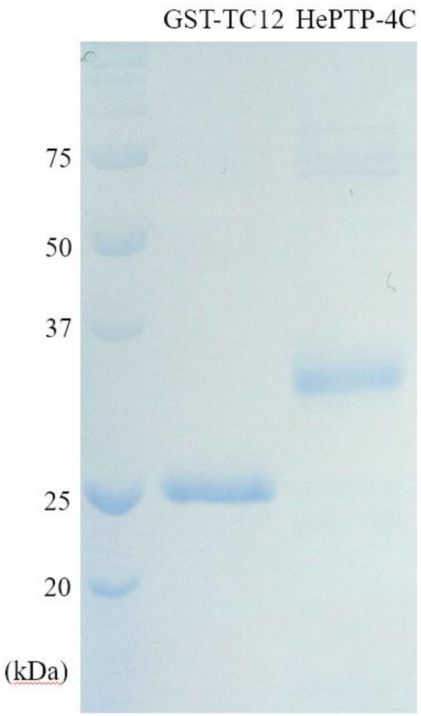

Figure S6. SDS-PAGE gel showing the purity of the proteins used in the study. The GST-TC12 has an expected mass of 27 kDa, whereas HePTP-4C is 35 kDa.

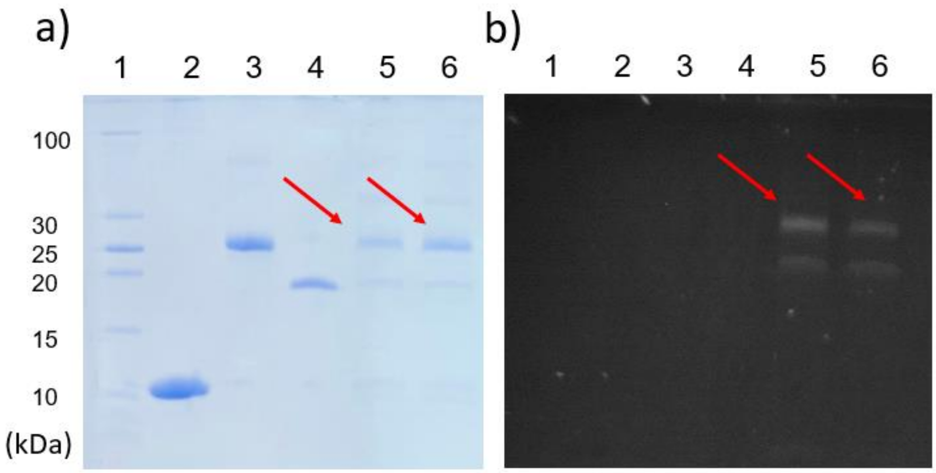

Figure S7. One-pot modification of glutathione S-transferase (GST-TC12) by synthesized probe with ubiquitin. GST-TC12 was added at molar ratios 1:1 and 3:1 relative to the probe. Samples: 1 – ladder, 2 – Ub-LPNTG, 3 – GST-TC12, 4 – sortase A, 5 – Ub-LPNTG + SrtCrAsH-EDT$_2$ + GST-TC12 (1 eq.), 6 – Ub-LPNTG + SrtCrAsH-EDT$_2$ + GST-TC12 (3 eq.). Reactions were analyzed by SDS-PAGE with visualization by (a) Coomassie staining and (b) fluorescent gel scanning. Red arrows indicate the main product (expecting mass ~37 kDa).

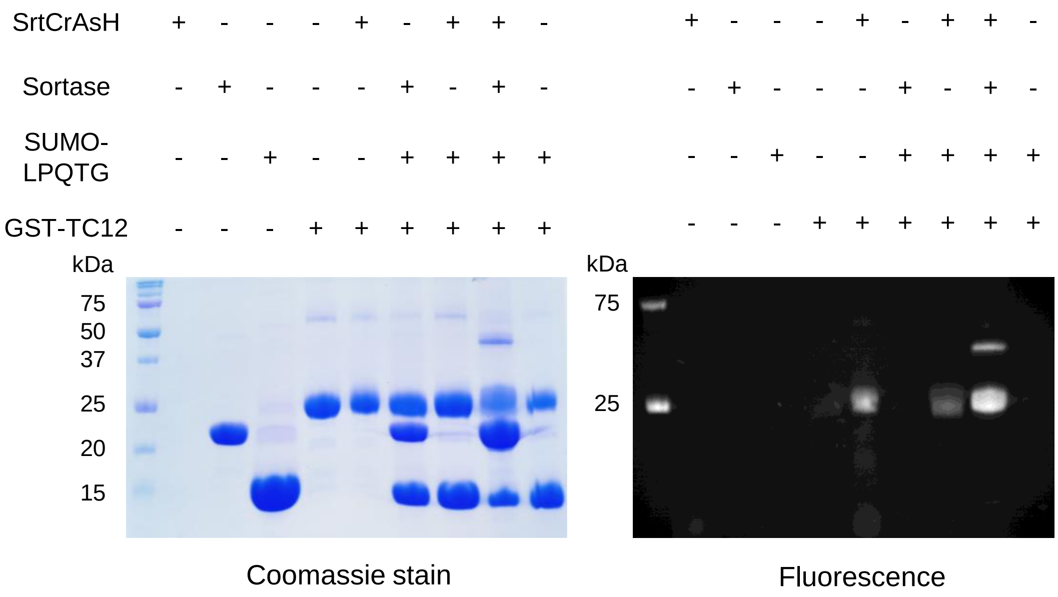

Figure S8. One-pot modification of glutathione S-transferase (GST-TC12) by SrtCrAsH with SUMO. Formation of the expected product of approximately 37 kDa is dependent on the presence of all ingredients of the reaction.

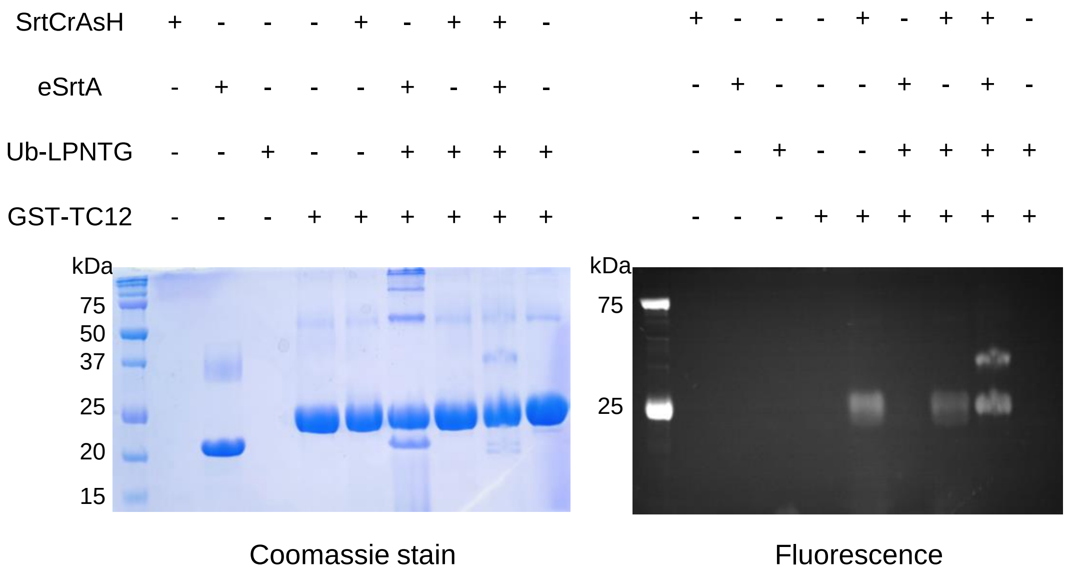

Figure S9. One-pot modification of glutathione S-transferase (GST-TC12) by SrtCrAsH with Ubiquitin. Formation of the expected product of approximately 37 kDa is dependent on the presence of all ingredients of the reaction.

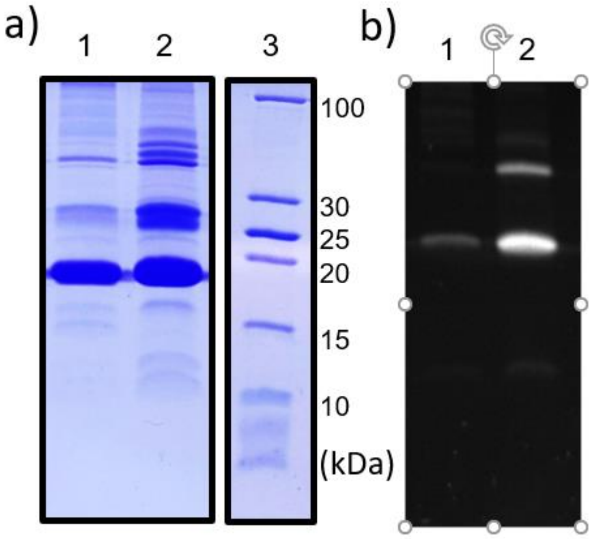

Figure S10. DeSUMOylation assay of the final SUMO-SrtCrAsH-GST conjugate using ULP1. The reaction was analyzed by SDS-PAGE with visualization by (a) Coomassie staining and (b) fluorescent gel scanning. Samples: 1 – reaction mixture of Ub-LPNTG, SrtCrAsH-EDT$_2$, and GST-TC12 after ULP1 treatment, 2 – reaction mixture of Ub-LPNTG, SrtCrAsH-EDT$_2$ and GST-TC12, 3 – ladder. The expected mass of the final product is ~38 kDa.

References

1. Martin, B. R.; Giepmans, B. N.G.; Adams, S. R.; Tsien, R. Y. Mammalian cell-based optimization of the biarsenical-binding tetracysteine motif for improved fluorescence and affinity. *Nat. Biotechnol*. **2005**, *23*, 1308−1314.
